# Understanding the impact of Achilles lipid content on tendon mechanical parameters: a cross-sectional study of people with familial hypercholesterolemia and healthy controls

**DOI:** 10.1186/s12891-025-08430-4

**Published:** 2025-02-22

**Authors:** Kipling Squier, Charlie Waugh, Joanne Callow, Wayne Patola, Michael A. Hunt, Liam R. Brunham, Jennifer Jakobi, Alexander Scott

**Affiliations:** 1 Department of Physical Therapy, Faculty of Medicine, University of British Columbia, Vancouver, BC Canada; 2 Centre for Aging SMART at VCH, Vancouver Coastal Health Research Institute, Vancouver, BC Canada; 3 School of Nursing, Faculty of Applied Science, University of British Columbia, Vancouver, BC Canada; 4 Department of Radiology, Faculty of Medicine, University of British Columbia, Vancouver, BC Canada; 5 Centre for Heart Lung Innovation, Faculty of Medicine, University of British Columbia, Vancouver, BC Canada; 6 School of Health & Exercise Sciences, University of British Columbia Okanagan, Kelowna, BC Canada; 7 Aging in Place Research Cluster, University of British Columbia Okanagan, Kelowna, BC Canada

**Keywords:** Familial hypercholesterolemia, Xanthoma, Cholesterol, Lipid, Achilles, Tendon, Biomechanics, MRI, Collagen

## Abstract

**Background:**

Familial hypercholesterolemia (FH) is a genetic condition that affects cholesterol metabolism, resulting in life-long elevated serum levels of low-density lipoprotein cholesterol. Systemically elevated cholesterol levels are associated with the onset of tendon injury and potentially lead to impaired mechanical properties. Applying a cross-sectional design, we examined whether FH patients present with altered Achilles biomechanics compared to healthy controls and conducted correlational analyses to determine the relationship between Achilles tendon biomechanics and tendon lipid or water content.

**Methods:**

Patients with FH (*n* = 33) and healthy controls (*n* = 31) were recruited from the Greater Vancouver area. Achilles cross sectional area, thickness, lipid and water content was determined using Dixon method magnetic resonance imaging (3.0T). Achilles mechanical properties were determined using synchronized dynamometry, motion capture, ultrasound and electromyography during ramped maximal voluntary isometric contractions, and stiffness and Young’s modulus calculated. Between group differences were assessed with independent t-tests or Mann-Whitney U tests and Pearson’s *r* or Spearman’s ρ were employed for correlational analyses. Sensitivity analysis was conducted on FH patients diagnosed with Achilles xanthoma and the remaining FH patients.

**Results:**

FH patients had significantly elevated Achilles total water content (*p* = 0.006), cross-sectional area (*p* = 0.006), and thickness (*p* = 0.019). No between-group differences were observed in any of the biomechanical parameters. In patients with FH there were significant positive relationships between tendon lipid or water content and tendon strain (ρ = 0.35, *p* = 0.046; *r* = 0.42, *p* = 0.02, respectively). No significant relationships were observed in control participants. In patients with FH, increased tendon cross-sectional area was associated with reduced stiffness (*r*=-0.371, *p* = 0.033) and increased strain (*r* = 0.48, *p* = 0.005). The presence of xanthoma was associated with increased Achilles dimensions (*p* < 0.05), total water content (*p* = 0.03), strain (*p* = 0.029), and decreased Young’s modulus (*p* = 0.001).

**Conclusion:**

Increased Achilles lipid and water content is associated with increased tendon strain in people with FH and the presence of xanthoma might indicate altered tendon mechanics. This study holds relevance for individuals with hypercholesteremia, as best management practices advocate for physical activity as part of a healthy lifestyle.

**Supplementary Information:**

The online version contains supplementary material available at 10.1186/s12891-025-08430-4.

## Introduction

Characterized by severely elevated serum low-density lipoprotein levels, familial hypercholesterolemia (FH) is one of the most prevalent genetic disorders – affecting ~ 1:300 people [1]. Unfortunately, around 90% of individuals with FH have not been diagnosed [2] and without correct treatment they are at risk for atherosclerosis and early onset of coronary heart disease [3]. Low-density lipoproteins (structures with high quantities of cholesterol) appear to have an affinity with glycosaminoglycans and proteoglycans [4], leading to permeation and accumulation within the elastic layers of arteries, eventually resulting in atherosclerosis [5]. Following a similar hypothesized pathway, serum low-density lipoproteins infiltrate tendon tissue, undergo oxidation, and are subsequently engulfed by macrophages, with these cells becoming trapped within the tendon’s extracellular matrix (ECM) [6]. In more severe cases, this can lead to areas of focal or diffuse thickening (likely driven by increased water content due to inflammation and edema [7]), known as tendinous xanthoma [8]. Tendinous xanthoma present with obstructed and disorganized collagen fibre alignment, along with dysregulated synthesis of collagenous and non-collagenous proteins [6, 9–11]. This manifestation within the tendon has been associated with increased risk of tendon injuries [12–14], likely due to compromised tensile integrity highlighted by reduced stiffness and ultimate strength [15, 16]. The Achilles tendon is one of the most common locations for intratendinous xanthoma formation [17] - which may be due to a propensity for low-density lipoproteins to bind to glycosaminoglycans [5], which are present in higher concentrations in energy saving tendons [18]. 

Until recently, biomechanical assessments of tendons with hypercholesterolemia have primarily been conducted in animal in vitro studies, which have produced relatively mixed results regarding stiffness, Young’s modulus, strain and ultimate stress [16, 19–21]. While these studies were able to replicate hypercholesterolemia environments, their applicability to human patients is very limited [22]. To address this paucity in research, we conducted a study measuring in vivo Achilles biomechanics during walking trials - we found that FH patients with tendon xanthoma had reduced stiffness, increased hysteresis, and altered loading patterns compared to controls [23]. However, we were not able to quantify the extent of cholesterol accumulation within the tendon, thus the severity of tendon xanthoma and its relationship to biomechanical outcomes were unknown.

Fortunately, magnetic resonance imaging (MRI) with the Dixon method has emerged as an effective technique for non-invasive quantification of tendon lipid content [24, 25]. This method induces in-phase and out-of-phase cycling of fat and water echoes, resulting in the generation of images solely depicting lipid or water through the addition or subtraction of these signals. Initially, the Dixon method was used to quantify triglyceride presence in lean tissues [25], but was recently validated to quantify low-density lipoprotein cholesterol accumulation in Achilles xanthoma [24]. This advancement enables the quantification of lipid content within human tendons; however, its application in correlating results with in vivo Achilles tendon biomechanics remains unexplored. Understanding how lipid accumulation affects tendon biomechanics in FH patients could enhance diagnostics and rehabilitation, potentially reducing tendon injury risk.

To address the significant lack of research in this area, the primary objective of this study was to investigate the effects of high cholesterol on in vivo Achilles mechanical properties in FH patients and compare results with healthy control participants (CP). We hypothesized that FH patients would exhibit increased Achilles tendon lipid and water content, leading to altered biomechanical parameters (decreased stiffness and Young’s modulus, and increased strain) compared to CP. The secondary objective was to evaluate whether Achilles tendon lipid or water content and tendon dimensions were associated with altered biomechanical properties. We theorized that FH patients with more severe tendon affliction - characterized by increased lipid or water content and larger dimensions - would have greater alterations in tendon mechanics. Lastly, we performed a sensitivity analysis to evaluate the impact of diagnosed xanthoma on the biomechanical outcomes within the FH group. This multi-faceted approach provides insight into the effects of hypercholesterolemia and tendinous lipid accumulation on tendon composition, structure, and function in FH patients, offering a foundation for future research and clinical protocols.

## Methods

For involvement in the current cross-sectional study, individuals were asked to partake in two data collection sessions: an MRI scan (University of British Columbia MRI Research, Vancouver, Canada) to determine Achilles tendon lipid and water content, dimensions, and moment arm length; and dynamometry (The Centre for Aging SMART at Vancouver Coastal Health, Vancouver, Canada) for Achilles tendon biomechanical estimation. Data collection sessions were spaced no longer than three weeks apart. Recruitment and data collection occurred continuously from January to October 2023. This study was conducted in accordance with the Declaration of Helsinki and was approved by the University of British Columbia Clinical Research Ethics Board (CREB H19-03071). All participants provided informed written consent prior to data collection.

### Recruitment and participants

FH patients were recruited from the British Columbia Familial Hypercholesterolemia Registry (Healthy Heart Program - Prevention Clinic, St. Paul’s Hospital, Vancouver, Canada). CP were recruited from Greater Vancouver and did not report any health-related conditions that may impact the outcomes of investigation. CP were recruited using purposeful sampling to closely simulate the FH patients average age and activity levels.

For inclusion, FH patients must have been diagnosed with probable (DLCNS of 6–8) or definite (DLCNS > 8) FH. All participants were required to be 19–55 years old at time of testing, moderately active, not pregnant, and have a BMI (body mass index) of < 35 kg/m^2^. Having a diagnosis of xanthoma was not an inclusion or exclusion criteria for this study. Participants were excluded if they had prior acute injuries to the AT, chronic conditions that may negatively influence musculoskeletal tissues and function, recent lower limb injuries, or participated in a structured resistance training program specifically targeting the triceps surae in the year preceding data collection. All participants must have met MRI safety requirements, determined by the MRI screening form and trained personnel. FH patients were initially screened with information available within the FH registry (age, location, Dutch Lipid Clinic Network Score (DLCNS), presence of comorbidities, listed as ‘active,’ consented for research purposes). If they met the above criteria, they were contacted with an initial contact letter sent through email or post, depending on their preferred contact type.

### Data collection

Participants completed a custom ‘demographics and health history’ questionnaire (supplementary Table 1) and Global Physical Activity Questionnaire [26]. Lipid profile (low-density lipoprotein cholesterol and total cholesterol) and current lipid-lower medication were recorded from the most recent FH Registry records for each FH patients. Physician diagnoses of tendon xanthoma, xanthelasma, and eruptive or palmar xanthoma - physical symptoms of hypercholesterolemia - were retrieved from participant records. CP clinical characteristics were not applicable.

### Tendon MRI analysis

**Fig. 1:**
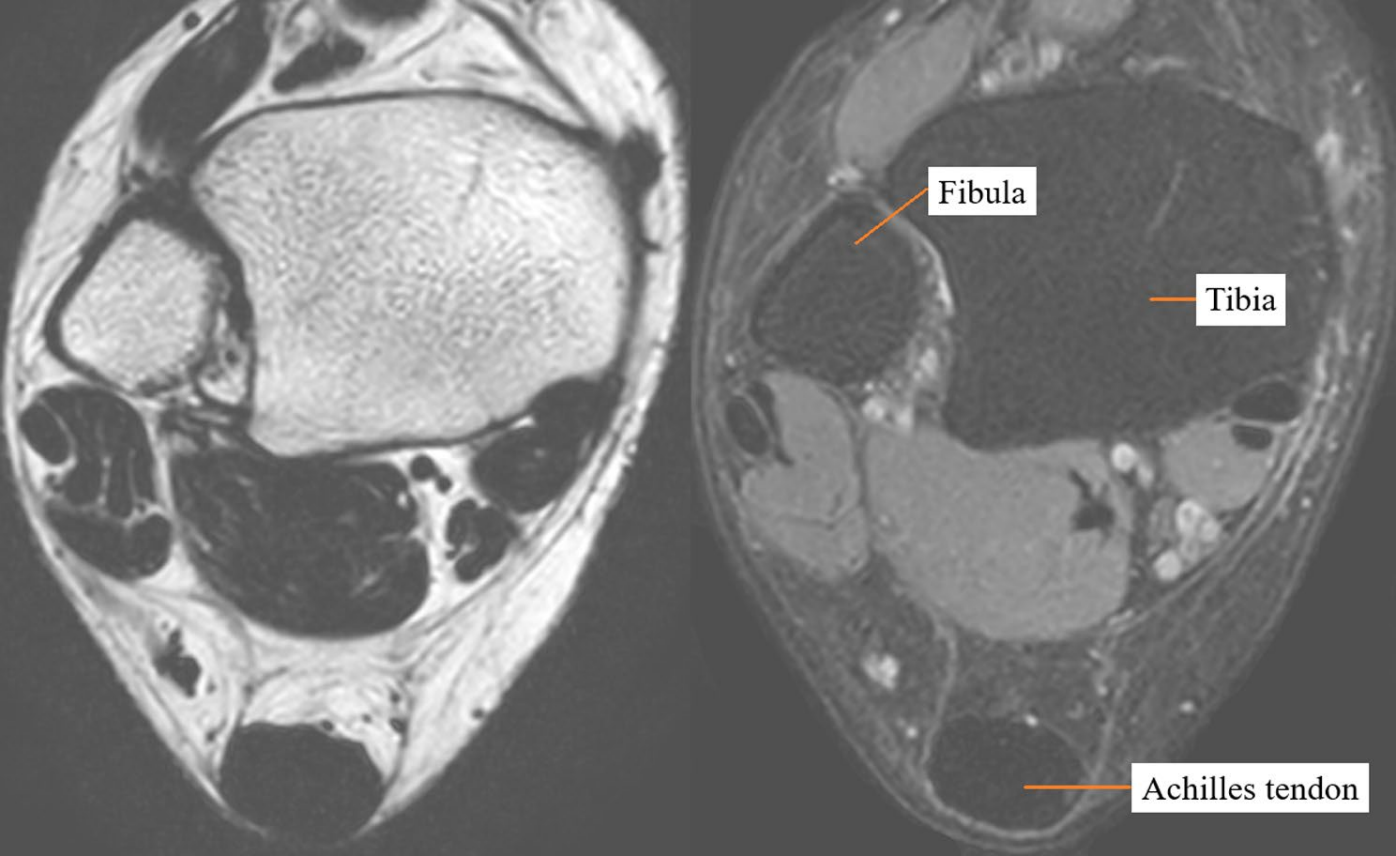
Dixon method MR imaging. Lipid-only (left), and water-only (right) sequences

Participants underwent MR scanning of their Achilles tendon bilaterally at the University of British Columbia MRI Research Centre. MRI protocols were performed by certified MRI technicians in a 3T MRI (Philips Elition, USA). Participants were requested to refrain from strenuous exercise 48 h prior to their MRI appointment. Participants were situated in the supine position on MRI examination table, with their ankle at a 90-degree angle in a transmit-receive ankle coil. One purified water and one oil (peanut) phantom were placed within the ankle coil, on the lateral and medial side of the participant’s heel, respectively. Participants underwent axial and sagittal scans, axial: proton-density turbo spin echo sequences utilizing a 3-point Dixon method in an 8 cm field of view (echo time 30; repetition time 3242; echo train length 6; frequency encodes 180 (interpolated to 448); phase encodes 175 (interpolated to 448); number of signal averages 1. 3 mm thick slices, with a 0.5 mm interslice gap for a total of 40 slices covering 13.95 cm with spatial resolution of 0.672 mm [3] (interpolated to 0.0972 mm [3]). Sagittal: proton-density turbo spin echo sequences also utilizing a 3-point Dixon method in a 12.8 cm field of view (echo time 30; repetition time 2500; echo train length 11; frequency encodes 256 (interpolated to 448); phase encodes 243 (interpolated to 448); number of signal averages 1. 2 mm thick slices with 0 interslice gap, for a total of 18 slices covering 3.6 cm with spatial resolution of 0.53 mm [3] (interpolated to 0.1682 mm [3]). A full scan procedure generated 4 sequences, consisting of in-phase, opposed-phase, lipid-only, and water-only sequences (Fig. 1). Originally, the Dixon method was utilized for triglyceride identification in lean tissues [25, 27, 28]. Since tendinous lipid accumulation is principally cholesterol, Griffith et al. validated methodology for use in patients with tendon xanthoma. They found exceptional correlation between cholesteryl esters and triglycerides (*r* = 1.0 [95%CI: 1.0, 1.0]) [24]. 

### Tendon biomechanical analysis

All biomechanical testing took place on the participant’s self-determined dominant leg. Participants were seated on an isokinetic dynamometer (Biodex 3, Medical Systems, USA) with their hip angle flexed to 85°, knee extended (180°), and neutral ankle position (0°) [29, 30]. This standardized position optimizes muscle length for maximal force production, which is less in a bent knee position due to its biarticular nature [31]. Participants had their foot securely strapped to the dynamometer footplate, with their malleolus aligned with the rotational axis of the dynamometer arm. To minimize body movement during contraction efforts, stabilization straps were placed across the torso, with arms crossed over the chest or folded on the lap.

Participants were made familiar with the testing protocol prior to set-up and were instructed to perform several submaximal contractions to acclimatize to plantarflexion torque development and pre-condition the tendon. This opportunity was used to instruct the participants to push through the ball of their foot and hinge at the ankle and avoid bending at the knee. Any incorrect muscle compensations or postures were addressed at this time. Participants were instructed to increase their contraction consistently until they hit their maximum (~ 2–3 s), then hold isometrically at their maximum torque production for 1–2 s, followed by a ~ 2–3 s ramp-down [32]. After acclimatization, participants completed three maximal voluntary contraction (MVC) trials (using the same instructions) with at least 1 min rest in between each trial. Participants were given verbal encouragement cues: “push, push”, “go, go, go”, “keep it up”. Trials were reperformed if torque generation was inconsistent or there were body positioning errors. Following the MVC trials, participants performed a single, ramped, sub-maximal dorsiflexion trial to quantify the antagonist coactivation of the tibialis anterior during the plantarflexion trial [33]. 

Displacement of the gastrocnemius medialis (GM) muscle-tendon junction (MTJ) during contraction efforts was measured with B-mode ultrasonography (Micrus ext-1 H, Telemed, Lithuania) using a 65 mm linear array probe (LV8-4L65s, Telemed) at 8 MHz scanning frequency, 38 Hz sampling frequency and maximum depth of 40 mm. The probe was fitted to a custom 3D printed housing, which was fixed (Transpore tape, 3 M, USA) over the GM MTJ on the participant’s dominant leg, medial to the septum with the gastrocnemius lateralis. The GM MTJ was digitized manually with Tracker (v6.0.0, physlets.org/tracker) to create 2D MTJ coordinates.

In conjunction with ultrasonography, an active motion capture system (Optotrak Certus, NDI, Canada) was utilized to obtain absolute Achilles tendon length. One marker was placed on the Achilles calcaneal insertion and three markers formed a rigid body on the probe housing. With the manufacturer digitizing probe, a virtual marker was created on the proximal edge of the probe’s scanning interface, derived from the rigid body marker coordinates. This virtual marker enabled the 2D MTJ coordinates to be transformed into the 3D motion capture reference frame and allow instantaneous tendon length and displacement to be obtained.

Analog data (plantarflexion ankle torque, electromyography and synchronization triggers) were sampled at 1000 Hz using a 16-bit A/D card (NI USB6225, National Instruments, USA) and DAQexpress software (v5.1, National Instruments). Plantarflexion ankle torque data were filtered using a low-pass second-order, zero-lag Butterworth filter with a 10 Hz cutoff frequency. Tibialis anterior electromyography data were collected using bipolar electrodes (Bagnioli-8, Delsys Inc., USA). Prior to electrode placement, skin was shaved, abraded, and cleaned for optimal electrode-skin contact and to lower impedance. The electrodes were positioned over the belly of the muscle, parallel to fascicular alignment. To reduce background noise, a reference electrode was placed on the participant’s patella. Electromyography signals were passed through a 20–450 Hz band-pass filter. Analog data were synchronized to motion capture and ultrasound data with synchronization triggers.

### Data analysis

#### MRI lipid and water signal analysis

**Fig. 2:**
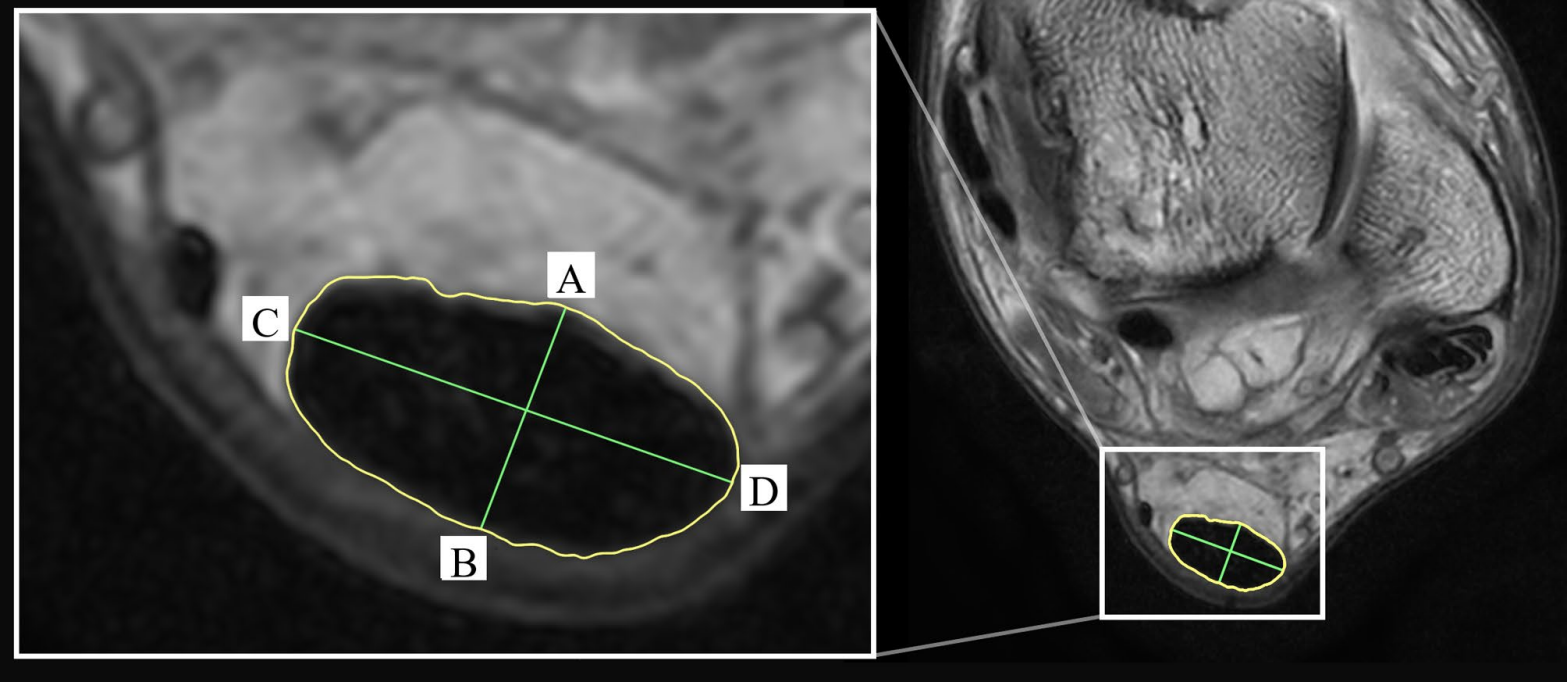
Contoured Achilles tendon with anterior-posterior thickness generation. Point **A** to point **B** represents anterior-posterior thickness. Line from point C to point D represents the greatest tendon width

MRI analyses were conducted using Horos MR imaging software (v4.0.1, Horos Project, USA) by a trained investigator who was blinded to group allocation. This investigator had previously displayed excellent intra-rater and inter-rater reliability using identical methods [34]. On the participant’s dominant leg, the investigator identified the slice with the greatest free Achilles tendon anterior-posterior thickness, defined as perpendicular to the longest width across the tendon, through the geometric center from the greatest anterior coordinates to the greatest posterior coordinates (Fig. 2). The tendon was manually contoured at this location, as well as the adjacent slice proximally and distally, for each of the in-phase, lipid-only and water-only sequences. Achilles tendon lipid and water signal intensity, cross-sectional area, and anterior-posterior thickness was collected. Relative lipid and water signal intensity was determined by contouring three consecutive slices of the respective phantoms, then dividing the tendon lipid and water values by the averaged phantom signal. Lipid (FH patients:1640 ± 180; CP:1665 ± 258.34) and water (FH patients:1714 ± 171; CP:1847 ± 162.34) phantom signals were relatively consistent across the groups. To achieve the total tendon lipid and water content (in arbitrary units), the relative lipid and water signals were multiplied by the cross-sectional area, as per Griffith et al. [24] and Zahradnik et al. [35]. All outcomes from each of the three slices were averaged to yield single outcome values.

Minimum free tendon cross-sectional area and Achilles tendon moment arm length were extracted from axial MR images and used for calculating tendon biomechanical properties (stress and tendon force, respectively). Achilles tendon moment arm length was calculated as the shortest distance from the ankle center of rotation to the middle of the Achilles tendon, representing the tendon line of action. Ankle joint center of rotation was designated as the coordinates equidistant between the lateral and medial malleolus.

### Achilles tendon force

The tibialis anterior displays antagonist coactivation during plantarflexion, resulting in a minor yet significant opposing torque. This leads to a reduction in the ankle torque recorded by the dynamometer, represented as net plantarflexion torque [33]. To account for this influence, tibialis anterior torque during each plantarflexion trial was estimated using the electromyography -torque relationship during the separate submaximal dorsiflexion trial performed by the participant following all MVC trials. This relationship is assumed to be linear during low levels of tibialis anterior coactivation [36]. The according tibialis anterior torque was added to the net recorded plantarflexion torque to yield gross plantarflexion torque. Achilles tendon force was calculated from the gross plantarflexion torque collected during MVC trials, divided by Achilles tendon moment arm.

$$\begin{aligned}&Gross\:AT\:Force\:\left(N\right)\quad=\frac{\left(\begin{aligned}&net\:plantarflexion\:moment\cr&\quad+tibialis\:anterior\:moment\end{aligned}\right)}{Achilles\:tendon\:moment\:arm}\end{aligned}$$

Rate of force development has been shown to influence tendon fascicle elongation [37], but the overall effect may be minimal [38]. Accordingly, we calculated rate of force development between 20 and 90% peak (N/sec) tendon force for group comparison.

### Achilles tendon deformation

Instantaneous Achilles tendon length (prior to contraction) was measured as the linear distance from the coordinates of the digitized GM MTJ to the coordinates of the Achilles insertion on the calcaneus, as defined by the motion capture marker. To determine intra-rater reliability (only one researcher performed the analysis), trials from three participants were randomly selected, and their MTJ coordinates were tracked three times per MVC. The coefficient of variation was calculated for each frame and averaged across the trial, yielding a mean variation of 1.73 ± 0.07%, demonstrating excellent reliability.

### Achilles tendon biomechanical outcomes

Achilles tendon strain (%) was calculated as the change in tendon length from rest to peak displacement. Achilles tendon stiffness (N/mm) was derived from a linear fit of the load-deformation relationship, between 20 and 90% of peak force. Stiffness within this force range provided an excellent fit (R^2^ = 0.943) and a mean coefficient of variation of 9% (calculated for each participant across their three trials and then averaged per group). Stress was calculated as force divided by the smallest cross-sectional area of the free tendon. Young’s modulus (MPa) was estimated slope of the tendon stress-strain relationship between 20 and 90% peak tendon stress.

### Statistical analyses

All statistical analysis was performed on SPSS software (v.25, IBM, Chicago). Significance was established at *p* < 0.05 for all statistical tests. Data were tested for normality and homogeneity of variances and with Shapiro-Wilk test and Levene’s test, respectively.

A sample size calculation using GPower (v3.1) was conducted for our primary objective (comparing biomechanical outcomes between groups) based on previous Achilles tendon stiffness data comparing FH patients with control participants during gait analysis [23]. This calculation determined an estimated sample size of 60 (d = 0.75, α = 0.05, 1-β = 0.80). To test the primary hypothesis, independent t-tests were used to test for group differences in Achilles tendon MRI and biomechanical outcomes; Mann-Whitney U tests were performed when normality was violated.

The secondary objective was to examine the relationships between Achilles tendon MRI outcomes (lipid or water content and Achilles tendon cross-sectional area or thickness) with Achilles tendon stiffness and strain, in both groups. The relationship between tendon lipid and water ratios was also assessed to determine whether it was possible to differentiate the relative impact of lipid or water content on tendon biomechanics. Pearson correlation coefficient (*r*) tests were used to determine relationships - Spearman’s rank correlation coefficient (*ρ*) was used when variables breached test assumptions.

Since xanthoma have been associated with altered tendon structure [6], we performed a sensitivity analysis to determine whether the diagnosed presence of Achilles xanthoma was an influential factor for MRI characteristics and biomechanical outcomes. Sensitivity analysis was conducted by comparing a subgroup of FH patients with documented xanthoma to FH patients without; Welch’s t-test was used to assess group differences in MRI characteristics and biomechanical outcomes.

## Results

35 FH patients were enrolled into the study alongside 35 CP. Data from 6 participants were excluded from all analysis due to MRI scan artifacts (2 FH patients and 2 CP), an error in plantarflexion torque collection (1 CP), and a failed day-of MRI screening (1 CP). Data from 64 participants (33 FH patients and 31 CP) were included in analysis, characteristics are shown in Table 1. FH patients demonstrated well moderated low-density lipoprotein cholesterol (2.81 ± 1.44 mmol/L) and total cholesterol (4.65 ± 1.51 mmol/L) profiles in their most recent (mean 23 ± 10 months) FH Registry records. Records indicated a median DLCNS of 9 with an interquartile range of 4.5, representing an average ‘definite FH’ diagnosis. Physical signs of FH (xanthoma) were reported in 8 FH patients’ records – xanthoma in the Achilles tendon (*n* = 5) xanthelasma (*n* = 3), and hand extensor tendons (*n* = 1), and palmar xanthoma (*n* = 1). All but four FH patients were currently using one or more medications prescribed for lipid management. Two CP reported left leg dominance, all other participants were right leg dominant.

**Table 1:** Participant characteristics

Characteristic	FH patients	CP
Participants (Female/Male)	33 (16/17)	31 (15/16)
Age (years)	41.7 ± 9	40.2 ± 9.1
Height (cm)	172.1 ± 10.3	173.1 ± 9.3
Body mass (kg)	79.6 ± 11.1	74.4 ± 13.2
BMI (kg/m^2^)	27 ± 3.8	25 ± 2.6
Activity level (MET mins)	2016 ± 1434	1993 ± 1079

Values are displayed as mean ± SD. BMI = body mass index; MET = metabolic equivalent of task

Achilles tendon cross-sectional area and anterior-posterior thickness were both significantly larger in FH patients, likely attributed to significantly elevated total water content (Table 2). Total Achilles tendon lipid content did not significantly differ between groups. In the axial view, the Achilles tendon mid portion took on an appearance of anterior concavity (crescent shape) in 80.6% of CP and only 48.4% of FH patients, the rest presented with anterior convexity.

**Table 2:** Achilles tendon MRI analysis characteristics

Outcome	FH patients	CP	*p*-value	Statistic	Effect size
CSA (mm^2^)	85.7 ± 28.2 [75.7–95.7]	69.7 ± 14.5 [66.4–75.0]	0.006^a^	*t =* 2.88	0.71
Tendon thickness (mm)	7.1 ± 1.9 [6.4–7.8]	6.0 ± 0.8 [5.7–6.3]	0.019^b^	*U =* 337	0.61
Total lipid content (AU)	3.8 ± 1.8 [3.1–4.4]	3.0 ± 0.9 [2.7–3.3]	0.089^b^	*U =* 385	0.44
Total water content (AU)	5.3 ± 2.3 [4.5–6.1]	3.9 ± 1.3 [3.4–4.4]	0.006^b^	*U =* 306	0.74
Lipid/phantom ratio	0.047 ± 0.011 [0.040–0.048]	0.043 ± 0.008 [0.040–0.046]	0.888^b^	*U =* 501	0.035
Water/phantom ratio	0.061 ± 0.013 [0.056–0.066]	0.055 ± 0.011 [0.051–0.059]	0.067^b^	*U =* 375	0.47

Group descriptives displayed as mean ± SD [95% CI]. *p*-values and test statistic obtained through ^a^independent t-tests or ^b^Mann-Whitney U tests. Effect size calculated as Cohen’s *d.* CSA = cross-sectional area; AU = arbitrary units

**Fig. 3:**
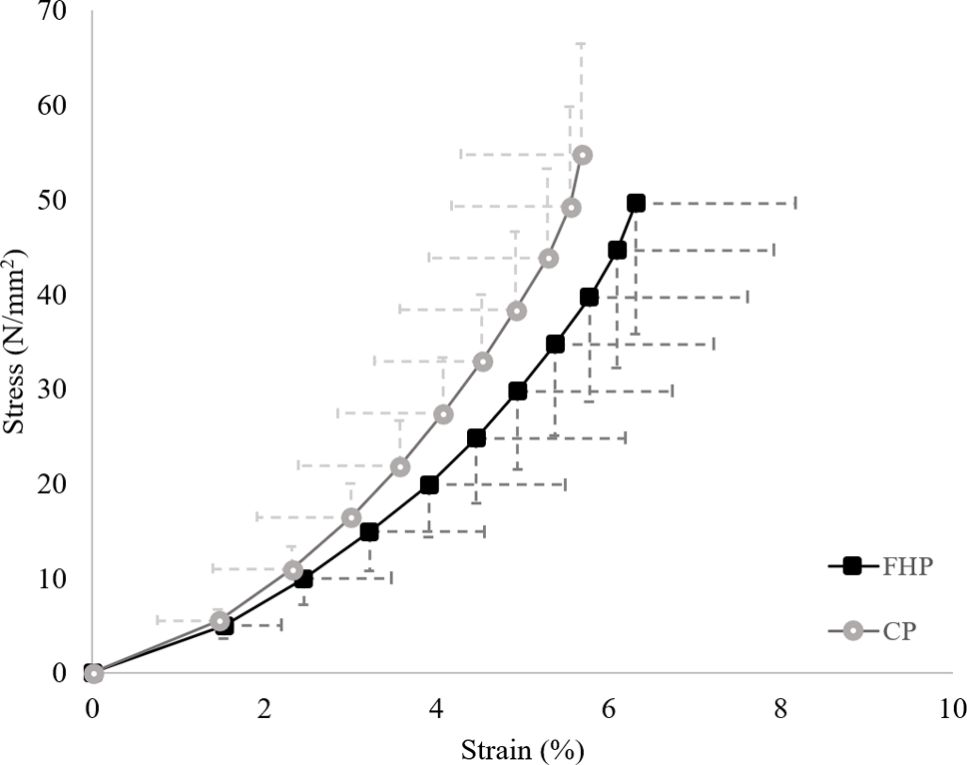
Group mean stress-strain curve from 0 to 100% stress. FH patients’ outcomes are characterized by black squares (dotted line) and CP with grey circles (solid line). Half error bars represent standard deviation of stress (vertical) and strain (horizontal) for each group. FHP = FH patients, CP = control participants

There were no between-group differences observed in any biomechanical outcome (Table 3). Tendon Young’s modulus trended toward a difference between groups (visualized as the stress-strain relationship, Fig. 3), but did not reach statistical significance. Peak tendon force and rate of force development were remarkably similar between groups. Achilles tendon moment arm (FH patients: 42.5 ± 4.1 mm; CP: 42.3 ± 2.9 mm) and Absolute tendon length (FH patients: 192.8 ± 26.26 mm; CP: 193.4 ± 30.5 mm) were also comparable.

**Table 3:** Achilles tendon biomechanical parameters descriptives and statistics

Outcome	FH patients	CP	*p*-value	Statistic	Effect size
Stiffness (N/mm)	373 ± 91 [341–405]	396 ± 76 [368–424]	0.282^a^	*t*=-1.09	0.27
Young’s mod. (MPa)	1017 ± 383 [881–1152]	1168 ± 276 [1067–1270]	0.075^a^	*t*=-1.8	0.45
Peak strain (%)	6.5 ± 1.9 [5.8–7.2]	5.9 ± 1.7 [5.3–6.5]	0.151^b^	*U* = 405	0.33
Peak stress (N/mm^2^)	49.6 ± 13.9 [44.7–54.5]	54.8 ± 11.8 [50.5–59.1]	0.113^a^	*t*=-1.6	0.40
Peak tendon force (N)	3559 ± 679 [3318–3800]	3511 ± 746 [3237–3785]	0.778^a^	*t*=-0.27	0.07
RFD (N/sec)	1395 ± 605 [1181–1610]	1418 ± 562 [1213–1625]	0.874^a^	*t*=-0.16	0.04

Group descriptives displayed as mean ± SD [95% CI]. *p*-values and test statistic obtained through ^a^independent t-tests or ^b^Mann-Whitney U tests. Effect size calculated as Cohen’s *d.* RFD = rate of force development

**Fig. 4:**
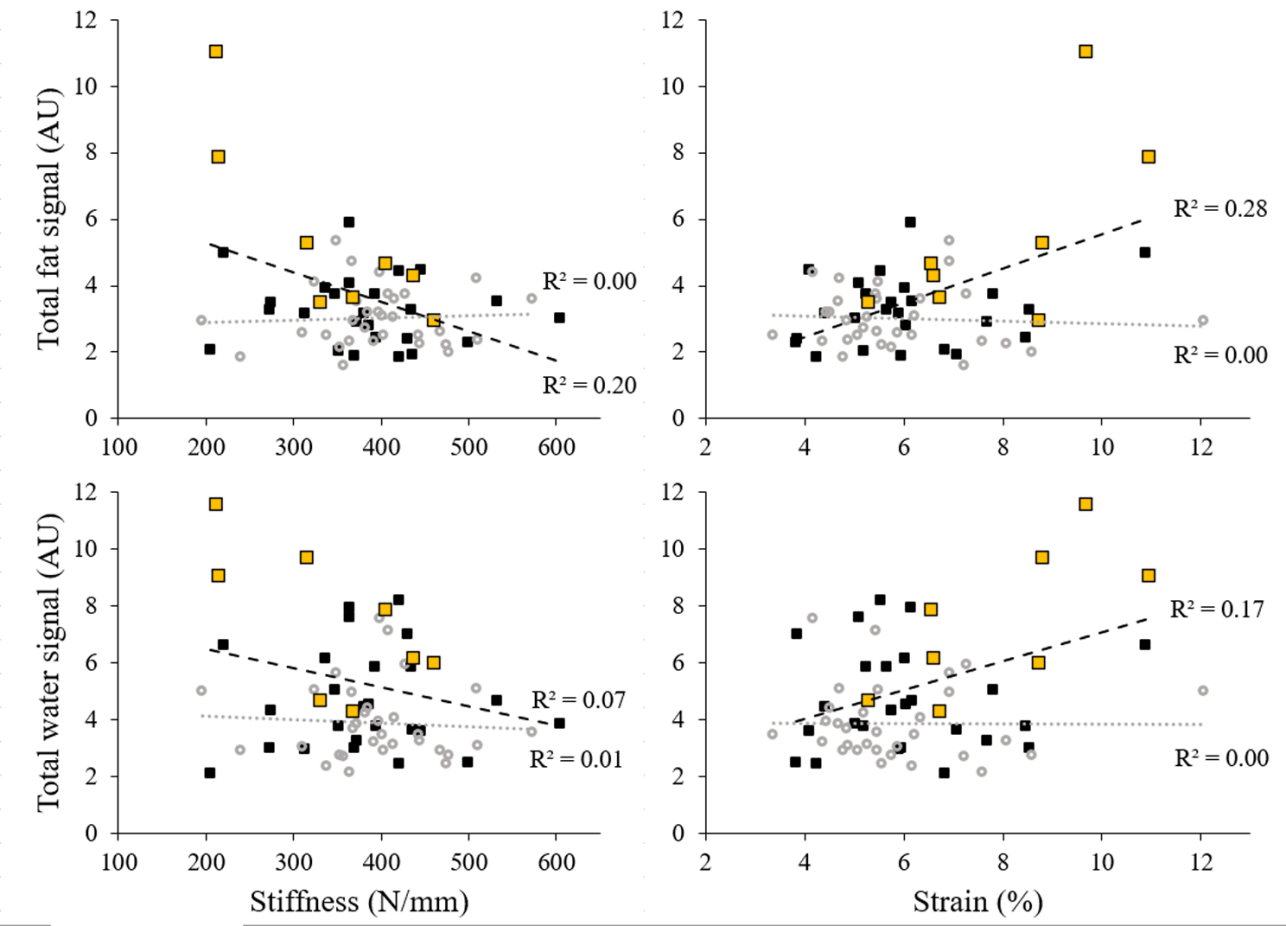
Relationship between total lipid or water content and stiffness or strain. Black squares and trendline represent FH patients’ outcomes, grey circles and trendline represent CP outcomes, yellow squares represent FH patients with xanthoma. No relationship between total lipid or water content and stiffness (FH patients: ρ=-0.31, *p* = 0.08, CP: *r*=-0.008, *p* = 0.97; FH patients: *r*=-0.27, *p* = 0.125, CP: ρ=-0.009, *p* = 0.962; respectively). No relationship between CP total lipid or water content and strain (ρ=-0.12, *p* = 0.518; ρ=-0.17, *p* = 0.362, respectively)

The total contents of both lipid and water were correlated with strain (ρ = 0.35, *p* = 0.046 and *r* = 0.42, *p* = 0.02, respectively) in FH patients, but not in CP (Fig. 4). In both groups, the total contents of neither lipid nor water were correlated with stiffness.

**Fig. 5:**
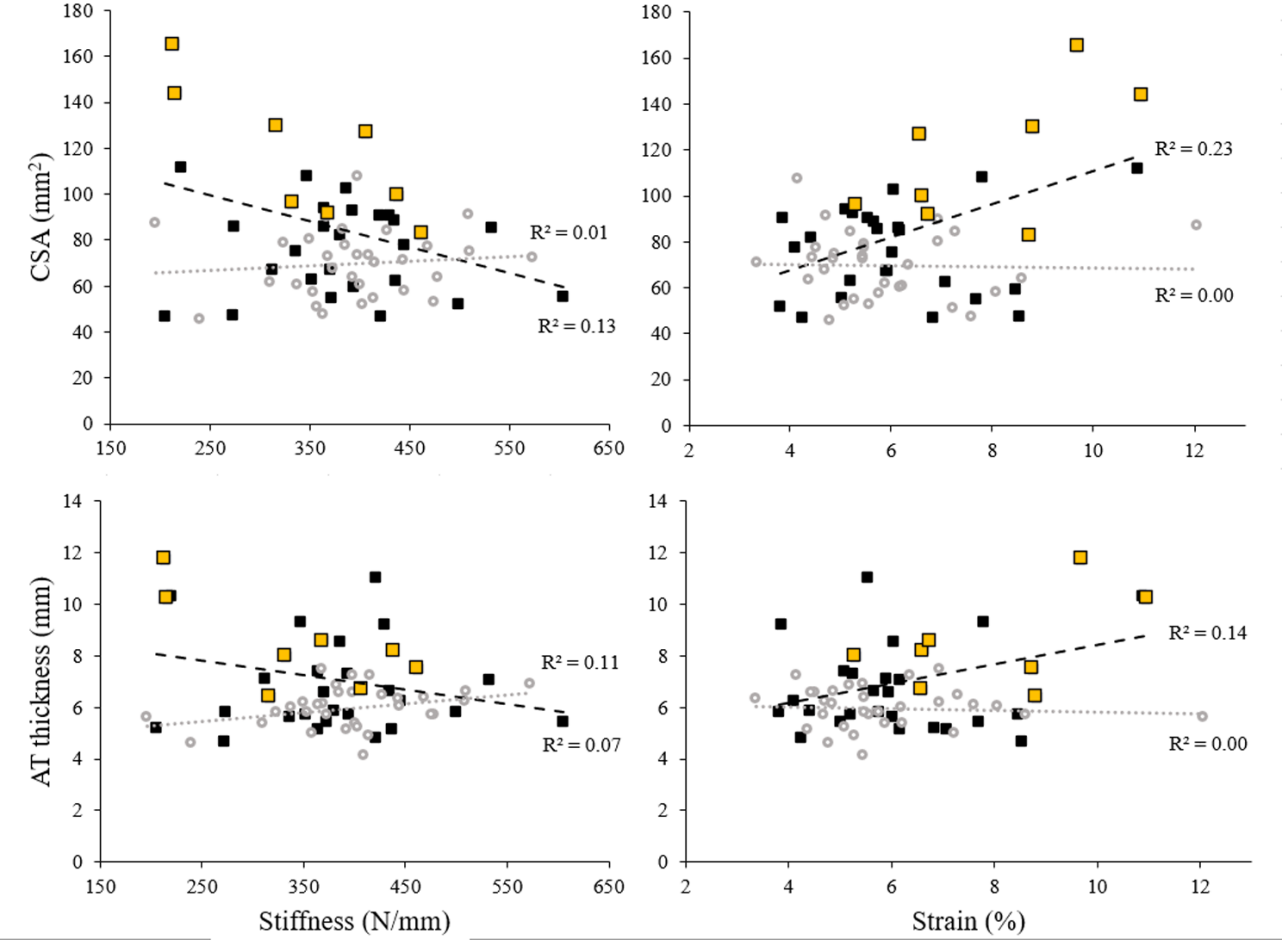
Cross-sectional area and Achilles tendon thickness relationships with Achilles tendon stiffness and strain. Black squares and trendline represent FH patients’ outcomes, grey circles and trendline represent CP, yellow squares represent FH patients with xanthoma

FH patients’ cross-sectional area correlated negatively with stiffness (*r*=-0.371, *p* = 0.033) and positively with strain (*r* = 0.48, *p* = 0.005); thickness did not have any relationship with the outcomes (Fig. 5). CP cross-sectional area and thickness did not correlate with either stiffness or strain. It was noteworthy that Achilles tendon thickness trended towards a positive relationship with stiffness (*r* = 0.32, *p* = 0.082) in this group. Lastly, we analysed the relationship between the lipid ratio and water ratio, which proved to be significant in FH patients (*r* = 0.366, *p* = 0.036) and CP (*r* = 0.476, *p* = 0.007).

Sensitivity analysis was performed by comparing MRI characteristics and biomechanical outcomes between a subgroup of FH patients with documented Achilles xanthoma and the remaining FH patients without documented physical signs. Analysis revealed that the xanthoma subgroup had increased cross-sectional area, thickness, and total water content, alongside elevated peak strain and reduced Young’s modulus and stress, Table 4. Characteristics (body mass, BMI, age, and activity level) did not differ between subgroups (all *p* > 0.05).

**Table 4:** FH patient xanthoma subgroup analysis of Achilles tendon MRI characteristics and biomechanical outcomes

Outcome	Xanthoma	No-xanthoma	*p*-value	Statistic	Effect size
Participants (female/male)	8 (4/4)	25 (12/13)			
CSA (mm^2^)	117 ± 29 [93–141]	75.7 ± 20 [68–84]	0.004	*t=*-3.767	1.85
Tendon thickness (mm)	8.4 ± 1.8 [6.9–9.9]	6.7 ± 1.7 [5.9–7.4]	0.034	*t=*-2.434	0.99
Total lipid content (AU)	5.4 ± 2.7 [3.1–7.7]	3.2 ± 1.0 [2.8–3.7]	0.062	*t=*-2.184	1.08
Total water content (AU)	7.4 ± 2.6 [5.2–9.5]	4.7 ± 1.8 [3.9–5.4]	0.02	*t=*-2.8	1.21
Stiffness (N/mm)	344 ± 93 [266–423]	384 ± 91 [346–422]	0.314	*t* = 1.053	0.44
Young’s mod. (MPa)	657 ± 258 [442–873]	1128 ± 340 [988–1270]	0.001	*t* = 4.145	1.46
Peak strain (%)	7.9 ± 1.9 [6.3–9.5]	6.0 ± 1.7 [5.3–6.7]	0.029	*t=*-2.606	1.09
Peak tendon force (N)	3880 ± 550 [3421–4340]	3456 ± 693 [3170–3742]	0.096	*t=*-1.778	0.64
Peak stress (N/mm^2^)	39.4 ± 8.5 [32.3–46.7]	52.5 ± 14 [46.7–58.3]	0.005	*t =* 2.462	1.01
RFD (N/sec)	1600 ± 613 [1088–2113]	1330 ± 599 [1083–1577]	0.297	*t=*-1.092	0.62

Group descriptives displayed as mean ± SD [95% CI]. *p*-values and test statistic obtained through Welch’s t-tests. Effect size calculated as Cohen’s *d.* CSA = cross-sectional area; AU = arbitrary units; RFD = rate of force development

## Discussion

The primary objective of the current study was to compare in vivo Achilles tendon mechanical properties in patients with FH compared to healthy individuals. We first quantified tendon lipid and water content in these groups using Dixon method MRI analysis and found significantly increased Achilles tendon water content and dimensions in FH patients. However, there were no between-group differences were observed in Achilles lipid content or biomechanics (stiffness, Young’s modulus and strain), which led to the rejection of our primary hypothesis. Importantly, the lack of differences in biomechanical variables was likely not attributable to the sample size, as power analyses indicated adequate statistical power to detect such differences. The secondary objective (relationship analysis between MRI and biomechanical outcomes) revealed that in FH patients, lipid and water content were positively associated with tendon strain, and cross-sectional area was positively associated with strain and negatively associated with stiffness, supporting our secondary hypothesis. No such associations were observed in CP. Sensitivity analysis within FH subgroups revealed that patients diagnosed with Achilles xanthoma exhibited increased tendon dimensions and water content, along with altered biomechanics characterized by decreased Young’s modulus and increased strain. These findings suggest that while FH patients may exhibit altered tendon morphology, biomechanical properties are not universally affected; the presence of Achilles tendon xanthoma is what appears to significantly influence these mechanical characteristics, potentially reducing the tendon’s ability to resist large loads. Understanding the physiological and biomechanical factors influencing tendon health is crucial for FH patients, given the well-documented association between elevated serum low-density lipoprotein cholesterol levels and tendinopathy development [12–14]. 

### MRI outcomes

Although attenuated, the results from MRI analysis were relatively consistent with prior studies [24, 35, 39]. We observed increased cross-sectional area, thickness, and total water content in FH patients. Unexpectedly, there was no difference in lipid content between the two groups. This could be attributed to prevalent usage of statins in FH patients, leading to well controlled lipid profiles and reduced likelihood of new lipid deposit formation within the tendon [40]. Statin therapies have been observed to regress tendon thickness in patients with FH without diffuse or focal tendon xanthoma, resulting from decreased lipid and water content [41]. Other researchers reported that statin treatment decreased tendon xanthoma lipid content, but also increased water content [24]. The authors theorized that mobilization and removal of trapped tendon lipids may result in incorporation of new hydrophilic ECM components and further inflammation [42, 43]. It seems that despite these adjustments of tendon lipid and water content, statins do not appear to influence collagen organization [44]. Regrettably, we were not able to comment on the effect of statins on tendon MRI characteristics or biomechanical parameters, as only four FH patients were not currently using any form of lipid lowering medication.

Consistent with our findings, water, rather than lipids, has been demonstrated to primarily contribute to the increased tendon cross-sectional area [24] and thickness [40] in FH patients, likely due to tendon water retention [11, 17] in areas of low grade inflammation [17]. This interaction is triggered by oxidized low-density lipoprotein molecules in the tendon ECM, where they are internalized by macrophages, leading to their transformation into large foam cells [45]. Foam cells become trapped in the ECM, resulting in substantial expression inflammatory cytokines [40]. Due to the influx of fluids into the tendon, the process ultimately leads to further low-density lipoprotein infiltration, altered tenocyte expression, and expansion of the tendon ECM [6]. These modifications may also create regions with poor collagen organization, content, and damage, contributing to increased thickness and decreased homogeneity within the collagenous matrix [10, 35]. Additionally, it is hypothesized that these cascading effects of cholesterol accumulation could impair tendon biomechanics and tendon healing, by interfering with the collagen matrix, and tenocyte mechanosensation and expression [11, 46]. However, despite our understanding of the fundamentals of cholesterol accumulation, the mechanisms through which this interaction can damage tendon collagen structures and alter composition remain relatively under-researched [9]. 

### Tendon biomechanics

Based on the findings of our previous FH gait analysis study [23], we anticipated significant between-group differences in outcomes relating to load-resistance, stiffness and Young’s modulus. However, we did not observe any between group differences in the Achilles biomechanical outcomes. This was interesting, as we theorized that cholesterol accumulation and the resulting pathological changes to the tendon’s ECM would adversely affect the tendon’s material properties. Young’s modulus trended lower in FH patients; this outcome is clinically relevant, as it is typically used to signify tissue resilience, a lower value could indicate reduced ultimate stresses, potentially leading to increased injury risk [47]. Prior investigations evaluating the effect of lipid accumulation on Achilles tendon Young’s modulus have only been conducted using ex vivo animal models and have produced mixed results. Waugh et al. reported no difference in patellar tendon stress or Young’s modulus in mildly hypercholesterolemic rats when compared to controls [21], while Grewal et al. reported reduced stresses and ultimate loads in mice with pronounced tendon cholesterol deposition [15]. This suggests that there could be an interaction between severity of cholesterol accumulation and altered tendon mechanics. Beason et al. also documented decreased Young’s modulus in hypercholesterolemic mice patellar tendons [16] and porcine bicep tendons [20]. Conversely, the same authors indicated modulus was significantly increased in high-cholesterol mice and monkey supraspinatus tendons [19]. Discrepancies in effects may stem from differences in species tendon structure [48, 49], physiology [50] and response to cyclic load [22], highlighting the inherent limitations of animal and ex vivo study designs. Further, the lack of definitive evidence could also stem from ex vivo study limitations, wherein tendon biopsies are hydrated to uniform levels for testing [51]. Our findings show significant tendon water content, which, as previously mentioned, may influence tendon mechanical behavior [52]. Ex vivo tendon hypercholesterolemia studies may yield more conclusive evidence if biopsies are able to be tested with water levels that are representative of their in vivo environment [19]. 

It is possible that certain factors contributed to the null findings - for example, we included ‘probable’ FH diagnosis in addition to ‘definite’ FH diagnosis, to enhance this studies applicability to the broader hypercholesterolemia population. Therefore, FH group in the current study exhibited a lower severity of disease (DLCNS: mean 9.8 ± 3.5) compared to our previous publication (DLCNS: mean 18 ± 6).^24^ The reduced severity in this sample may have attenuated the biomechanical manifestations of hypercholesterolemia, limiting the chance of detecting significant group differences in tendon stiffness and Young’s modulus. The difference in DLCNS was expected, as we previously required ‘definite’ FH and tendon xanthoma diagnosis, which likely led to more severe Achilles tendon lipid and water accumulation [35], altered collagenous matrix alignment and composition [9], and result in finding reduced tendon stiffness [23]. Further, the absence of an observed effect may stem from methodological differences in data collection activity (ramped MVC versus walking), provoking altered load-deformation relationships between studies. Previous research has demonstrated significant influences of loading and strain rates (elevated in walking trials compared to ramped MVCs) on the viscoelastic properties of tendons, having a stiffening effect when increased [53–55]. However, increased water retention may have modified this effect, leading to relatively decreased modulus [51, 52] due to enhanced inter-fibril and -fiber sliding from reduced friction [56]. This may have favored the previous study’s methodology in detecting a group difference. Similarly, our MVC protocol involved relatively slowly ramping (~ 2–3 s) to ensure a similar rate of force development across participants. This slower ramping rate may have allowed for greater water diffusion [57], thereby attenuating between group differences in water content and Achilles tendon viscoelastic properties [58].

### Relationships between MRI characteristics and biomechanical parameters

We observed positive correlations between Achilles tendon lipid or water content and Achilles tendon strain in FH patients. It is worth noting that fat and water ratios were moderately correlated, so differentiating the biomechanical effect of lipid verses water content was not possible with our statistical analysis. Regardless, the associations between tendon content and biomechanical parameters underscore the relationship between severe lipid and water infiltration and greater tendon extension, potentially rendering the Achilles tendon more susceptible to strain-based damage [59]. Similar to tendinopathic lesions [60], lipid accumulation inadvertently increases the prevalence of poorly aligned collagen [8] and collagen type III content [9] (a variant that is less resilient to tensile forces), contributing to altered extension mechanisms [61]. Prolonged elevated strains can lead to microscopic disruptions in the collagen network that foster a cycle of altered cellular expression [62], predisposing the tendon to tendinopathy or rupture [7, 60]. Although elevated water content may contribute to increased strains through greater fascicular sliding [51], the effect on injury risk may not be straightforward. Increased sliding could shield collagen fibers from stretch- and friction-based damage, influencing tendon resilience in unanticipated ways [63]. 

In gait application, increased tendon strain may affect energy transfer and recoil [59] – where the optimal force-length relationship of the triceps surae may be impacted [59, 64]. This concept was illustrated in our previous publication where we observed that patients with FH demonstrated increased energy lost over walking stance phase; specifically, we observed different loading behaviours early in the stance phase, which may have been a result of altered muscle-tendon unit functioning [23]. Excess tendon elongation could necessitate greater muscle fascicle shortening, increasing the metabolic cost of ambulation [65]. Although we were unable to directly assess this interaction in the current study, the data support the idea that tendon lipid and water accumulation may affect tendon extension, potentially leading altered viscoelasticity during movement. However, further investigation is required to explore this avenue more thoroughly.

Clinically, determining the amount of Achilles tendon water and lipid is not economically feasible. Since practical identification of tendon thickening can be achieved with MRI [66], ultrasound [39] and, to a lesser extent, physical inspection [40], we analyzed the relationship between Achilles tendon cross-sectional area or thickness and stiffness and strain outcomes as part of our second objective. Data revealed that FH patients’ cross-sectional area correlated negatively with stiffness, and positively with strain – suggesting that clinical identification of severe tendon expansion may indicate suboptimal tendon mechanics in individuals with hypercholesterolemia disorders. This phenomenon was not observed in CP, where a trend towards a positive relationship between thickness and stiffness was observed. This aligns with expectations in non-pathogenic tendons, where hypertrophy is typically attributed to increased collagen synthesis and integration - resulting in increased stiffness and Young’s modulus [67] and decreased risk of injury [68]. This contrast underscores the unique adaptations in FH patients’ tendons, where hypertrophy appears to reflect degenerative changes rather than functional improvements. These findings highlight the importance of targeted clinical assessments to inform rehabilitation strategies aimed at addressing compromised mechanics and reducing injury risk in people with severe tendinous cholesterol accumulation.

### Sensitivity analysis

We performed sensitivity analyses with a subgroup of FH patients with documented Achilles xanthoma to further evaluate the impact of xanthoma-based signs of FH on Achilles tendon parameters. The observed increases in Achilles tendon cross-sectional area, thickness and total water content among the xanthoma subgroup aligns with expectations since xanthoma diagnosis with physical examination typically requires visible or palpable tissue enlargement. While stiffness was not different between subgroups, Young’s modulus was significantly lower resulting from reduced stress values. Achilles tendon strain was also significantly higher in the xanthoma subgroup, supporting our hypothesis that the disorganization and compositional differences in xanthoma [6], along with increased total water content, may influence the tendon’s extension mechanisms. Like the above correlational analysis, this investigation suggests that severity of tendon affliction may be an indicator of altered tendon mechanics, which extends beyond the presence of FH alone. Clinical diagnosis of xanthoma may be underreported due to inconclusive physical examinations [40], prioritization of other outcomes, or virtual appointments - which also may have restricted the xanthoma subgroup sample size. Similarly, there could be individuals with undiagnosed xanthoma in the other subgroup, which may have attenuated subgroup differences. It is also difficult to ascertain whether these results are clinically relevant - observed differences in tendon properties may not be substantial enough to increase risk of injury or modify movement patterns. Therefore, conclusions based on these results should be approached with caution.

### Future directions

Future investigations could focus on determining whether MRI characteristics are proxies for altered biomechanical parameters using robust modeling methodologies. Additionally, research could investigate whether physical therapy interventions can normalize biomechanical and imaging outcomes in participants with more extreme values, as observed in the correlation analysis. Resistance-based exercise therapy is one of the more prevalent and successful strategies for managing chronic tendon disorders [67]. Appropriate loading protocols have been shown to stimulate tenocyte collagen synthesis and MMP activity [69] to remodel hypoechoic regions [10] and increase tendon stiffness and Young’s modulus [70, 71]. In theory, these modifications could reformat areas of diffuse or focal thickening and to normalize ECM structure.

### Limitations

The Achilles tendon length was determined with a straight-line approach, which does not account for the anterior curve of the tendon, likely underestimating Achilles tendon length and overestimating strain for all participants [72]. Although errors associated with this commonly used approach are assumed to be systematic in nature and would have minimal influence on our analysis this limitation requires recognition. To calculate stress the cross-sectional area was measured at different locations and Dixon method signal analysis used. Stress measurements are typically made using the smallest tendon cross-sectional area to exhibit the greatest stress concentration [73]. On the other hand, cross-sectional area for signal analysis was determined by contouring the slices surrounding the tendon’s greatest anterior-posterior thickness, which would be representative of areas with increased water or lipid signal density and may not necessarily align with the region of maximum stress concentration [67]. We recruited patient participants from a FH registry, which may have led to attenuated lipid profiles and severity of lipid or water infiltration relative to the undiagnosed and undertreated hypercholesterolemia population due to the level of care available to those in the FH Registry.

## Conclusion

We evaluated the impact of FH on Achilles tendon MRI characteristics and biomechanical parameters. Despite reporting significant increases in Achilles tendon cross-sectional area, thickness, and water content in people with FH, we did not detect between-group differences in the biomechanical outcomes. There were positive correlations in FH patients between total Achilles tendon lipid or water content and strain, suggesting altered tendon extension mechanisms and muscle-tendon unit force-length relationships that may increase risk of strain-based injury. We also found that Achilles cross-sectional area in FH patients was negatively correlated with stiffness and positively correlated with strain, suggesting that clinical assessment of tendon enlargement may serve as an indicator of altered tendon mechanics, warranting further investigation. Subgroup sensitivity analyses of FH patients indicated connections between xanthoma presence and reduced stiffness and Young’s modulus, respectively. Interpretation of these results enables speculation that severity of tendon expansion attributed to lipid and water content, rather than presence of hypercholesterolemia, may serve as an indicator for altered tendon mechanics. Our findings provide the basis for future research to investigate clinical interventions aimed at restoring normal tendon phenotype and tendon mechanics, in severe cases of hypercholesterolemia basted tendon pathology.

## Electronic supplementary material

Below is the link to the electronic supplementary material.

Supplementary Material 1

## Data Availability

The datasets generated and/or analyzed during the current study are available in the OSF repository: https://osf.io/btq9u/?view_only=3b143e0a5bfd4854b8235502637dcb84.

## Abbreviations

CP: Control group
DLCNS: Dutch Lipid Clinic Network Score
ECM: Extracellular matrix
FH: Familial hypercholesterolemia
GM: Gastrocnemius medialis
MRI: Magnetic resonance imaging
MTJ: Muscle tendon junction
MVC: Maximal voluntary contraction
