# Supplementary material for: Understanding the impact of Achilles lipid content on tendon mechanical parameters: a cross-sectional study of people with familial hypercholesterolemia and healthy controls
Source: BMC Musculoskelet Disord. 2025 Feb 22;26:183. doi: 10.1186/s12891-025-08430-4 (PMC11846310; doi:10.1186/s12891-025-08430-4)
Supplement: Supplementary file 1 — Supplementary Material 1 [file 12891_2025_8430_MOESM1_ESM.pdf]

Supplementary table 1. FHAT Participant Screening and Health History Questionnaire

Study ID:	Appointment time/date
Study Visit: Biodex / MRI	Completed GPAQ Yes / No
Completed MRI screening Yes / No	Age (years/months) on test day:
Sex: M / F	Standing Height:
Body Mass:	Dominant leg
*No Study data will be collected until informed consent is obtained.<br>*Questionnaire to be implemented by research staff.	

1	(FH participants) Have you ever experienced AT pain not attributed to acute injury? Explain.	
2	Have you experienced acute or chronic musculoskeletal disorders in the past year? Examples include tendinopathies and Hagland's deformity.	
3	Have you taken oral corticosteroids within the past year? Fluoroquinolones or quinolone medication family?	
4	Have you ever been diagnosed with diabetes?	

5	Have you ever been diagnosed you have any form of arthritis?	
6	Are you aware of any genetic disorders you have (other than FH) that may affect MSK? Example: Marfan syndrome, cerebral palsy.	
8	Have you exercised in the last 48 hours? If yes, what did you do?	
11	Smoking history?	
If FH participant indicated pain, use following questions.		
12	AT pain symptom duration?	
13	History of past tendon injury/pain at AT? Other tendon sites?	
14	Location of AT pain symptoms?	

15	Were any interventions used to aid it in the relief or treatment of the AT symptoms?	
